# Neuronal networks with NMDARs and lateral inhibition implement winner-takes-all

**DOI:** 10.3389/fncom.2015.00012

**Published:** 2015-02-18

**Authors:** Patrick A. Shoemaker

**Affiliations:** Tanner Research, Inc.Monrovia, CA, USA

**Keywords:** winner-takes-all, NMDA receptors, lateral inhibition, bistability, multistability, neural computation

## Abstract

A neural circuit that relies on the electrical properties of NMDA synaptic receptors is shown by numerical and theoretical analysis to be capable of realizing the winner-takes-all function, a powerful computational primitive that is often attributed to biological nervous systems. This biophysically-plausible model employs global lateral inhibition in a simple feedback arrangement. As its inputs increase, high-gain and then bi- or multi-stable equilibrium states may be assumed in which there is significant depolarization of a single neuron and hyperpolarization or very weak depolarization of other neurons in the network. The state of the winning neuron conveys analog information about its input. The winner-takes-all characteristic depends on the nonmonotonic current-voltage relation of NMDA receptor ion channels, as well as neural thresholding, and the gain and nature of the inhibitory feedback. Dynamical regimes vary with input strength. Fixed points may become unstable as the network enters a winner-takes-all regime, which can lead to entrained oscillations. Under some conditions, oscillatory behavior can be interpreted as winner-takes-all in nature. Stable winner-takes-all behavior is typically recovered as inputs increase further, but with still larger inputs, the winner-takes-all characteristic is ultimately lost. Network stability may be enhanced by biologically plausible mechanisms.

## Introduction

The winner-takes-all (WTA) function is an operation that is often assumed to take place in biological nervous systems, and it has been demonstrated to be a powerful computational primitive (Maass, 2000). Over the years, winner-takes-all networks have been modeled widely in the fields of computational brain science and artificial neural networks (Amari, 1972; Grossberg, 1973; Koch and Ullman, 1985; Rumelhart and Zipser, 1987; Yuille and Grzywacz, 1989; Coultrip et al., 1992; Ermentrout, 1992; Winder, 1999; Yuille and Geiger, 2003; Mao and Massaquoi, 2007; Handrich et al., 2009; Chen et al., 2013), and have also been implemented in various analog electronic circuits (Lazzaro et al., 1989; Andreou et al., 1991; Deweerth and Morris, 1995; Lau and Lee, 1998; Fish and Yadid-Pecht, 2001; Indiveri, 2001; Baishnab et al., 2010). These mathematical and silicon models for the most part achieve the WTA characteristic using some sort of common inhibitory feedback in conjunction with high gain and a strong nonlinearity, often in the form of a neural thresholding operation. In its strictest forms, the winner-takes-all amounts simply to selection of the largest from among a set of input signals; in “soft” implementations, it involves strong amplification of the difference between the largest and other such signals, with significant suppression of weaker signals within the set. Depending on the model, it may retain some analog information about the winning signal.

In this paper, I demonstrate a mechanism by which a neuronal network with global lateral inhibition, arranged in perhaps the simplest possible topology, is capable of realizing winner-takes-all. Although nonlinearities such as neural thresholding contribute to this capacity, it depends most critically on the unique electrical properties of *NMDA receptor* (NMDAR) ion channels, which are assumed to mediate excitatory input to the network. The NMDAR is a class of glutamatergic receptor for which N-methyl-D-aspartate (NMDA) is an agonist, and is found in many phyla and frequently associated with synapses. The current-voltage relationship of NMDAR ion channels is nonmonotonic under physiological conditions (Nowak et al., 1984; Jahr and Stevens, 1990), with a negative slope conductance regime due to (kinetically fast) magnesium blockade. This characteristic renders the NMDAR capable of supporting neural amplification (Shoemaker, 2011) and bistability (Lazarewicz et al., 2006; Shoemaker, 2011; Sanders et al., 2013) in conjunction with other membrane conductances. The primary finding here is that a WTA characteristic can be *induced* by high-gain regimes that result from such interactions, rather than requiring a high intrinsic or parametric gain in the feedback loops. In this respect, the model contrasts with other biologically-inspired WTA network models that in some way incorporate NMDARs (Winder, 1999; Handrich et al., 2009; Chen et al., 2013). It is significant because it represents a mechanism for WTA that is both simple and at the same time entirely plausible biophysically, relying on known characteristics of ubiquitous classes of synaptic receptors. It is also of interest due to the widespread distribution of neurons with glutamatergic synapses and lateral inhibition in many areas of the brain.

## Results

### The WTA network model

**Figure 1:**
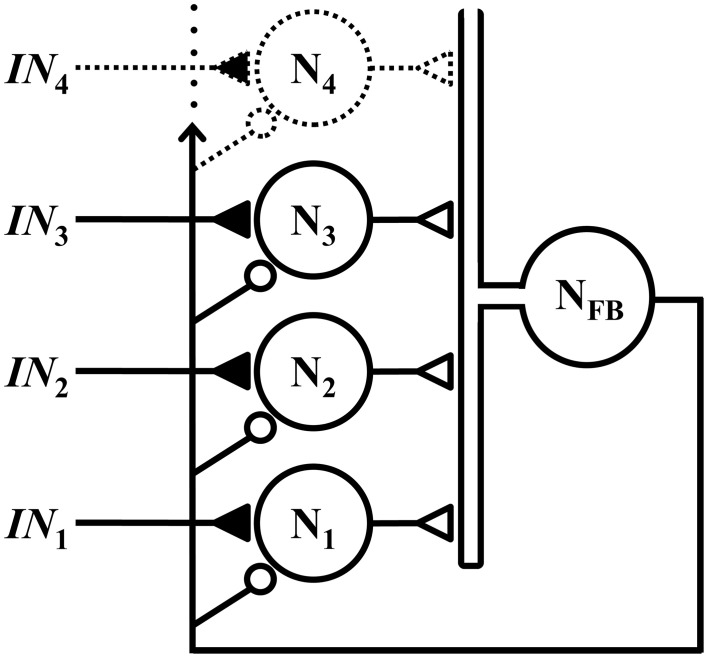
**Members of a set of competitive neurons N_1_, N_2_, N_3_, … each receives a respective excitatory input *IN*_1_, *IN*_2_, *IN*_3_, …, via an NMDA synapse (closed triangle)**. These neurons form excitatory synapses (open triangles) onto a common interneuron N_FB_, which projects feedback to all neurons in the set via inhibitory synapses (small open circles). Although not depicted, the outputs of neurons N_1_, N_2_, N_3_, … are assumed to also project to other parts of the nervous system.

The WTA microcircuit described herein assumes a classical lateral inhibitory topology, with a set of competitive neurons that receive excitatory inputs via NMDA synapses, and global feedback inhibition via a common interneuron, as illustrated schematically in Figure 1.

The WTA characteristic in this model is conceived as resulting from strong amplification and nonlinear effects that can emerge from interactions between NMDARs and the other ion channels in the membrane (in this case, the parallel resting and inhibitory synaptic conductances). These can result in an electrically bistable membrane, in which the dependence of the transmembrane current on the membrane potential shows the classical N-shape that is associated with destabilizing first order and stabilizing higher odd-order terms in a canonical bistable system (illustrated in **Figure 8** in the Methods Section). Such behavior is enhanced when the total non-NMDAR conductance has a reversal potential below typical neural resting potentials, and also when its current-voltage dependence is sub-linear or inward-rectifying (Shoemaker, 2011; Sanders et al., 2013). With this in mind, consideration is given to inhibitory feedback with several different characteristics: either mildly hyperpolarizing (e.g., as might be the case if chloride channels were associated with the inhibitory synapses) or more strongly hyperpolarizing (which might be expected with potassium channels), and having either ohmic or inward-rectifying channels. Gamma-aminobutyric acid (GABA)-mediated synapses, which are prime candidates to implement the inhibition, occur in two major classes that reflect different combinations of these characteristics: the GABA_B_ class involves Kir channels, which conduct potassium and are inward-rectifying (Mott and Lewis, 1994; Sodickson and Bean, 1996; Kaupmann et al., 1998; Fowler et al., 2007) (and thus might be expected to promote high-gain behavior), whereas GABA_A_ receptor channels conduct chloride and are non-rectifying (Johnston, 1996; Olsen and DeLorey, 1999).

Development of a mathematical model (described in the Methods section) and numerical analysis are useful tools for analyzing the range of behaviors than can be expected from such a network. The model used herein is based on simple isopotential neurons. Neural signals are represented as continuous in time, with any spiking behavior assumed representable by mean spike rates that are in turn related to neural depolarization. Several different inhibitory characteristics are considered, including ohmic and inward rectifying channels with either weakly or strongly hyperpolarizing reversal potentials, in order to illustrate a range of behaviors associated with plausible inhibitory mechanisms. Magnitudes of synaptic conductances are specified in the text relative to resting membrane conductance. In addition to the inhibitory reversal potential, free parameters in the model include the magnitudes of the inputs to the network, and a *loop gain* parameter (defined in Methods) that applies to the inhibitory feedback. The instantaneous loop gain (i.e., the incremental gain around a feedback loop) of any particular circuit is state-dependent and can greatly exceed this loop gain constant in magnitude, but as a parameter it accounts for the strengths of the input and output synapses of the inhibitory interneuron and thus is useful to quantify the effectiveness of the feedback.

### Analysis of stationary equations

Although in a biological system such a network would be expected to operate under dynamic conditions, a stationary analysis—i.e., determination of the fixed points of the model's governing equations—can give significant insight into its functional characteristics. I undertake such an analysis in this section to characterize the range of stationary behaviors that can be expected, and in particular to determine the existence of multiple fixed point solutions for given levels of synaptic input. In the absence of time-dependence in the feedback loops, such solutions are indicative of bi- or multi-stable regimes, and I use these terms to refer to them throughout this section. However, whether such fixed points *are* in fact stable depends on the dynamical characteristics of the feedback pathway, which will be considered in the following section.

**Figure 2:**
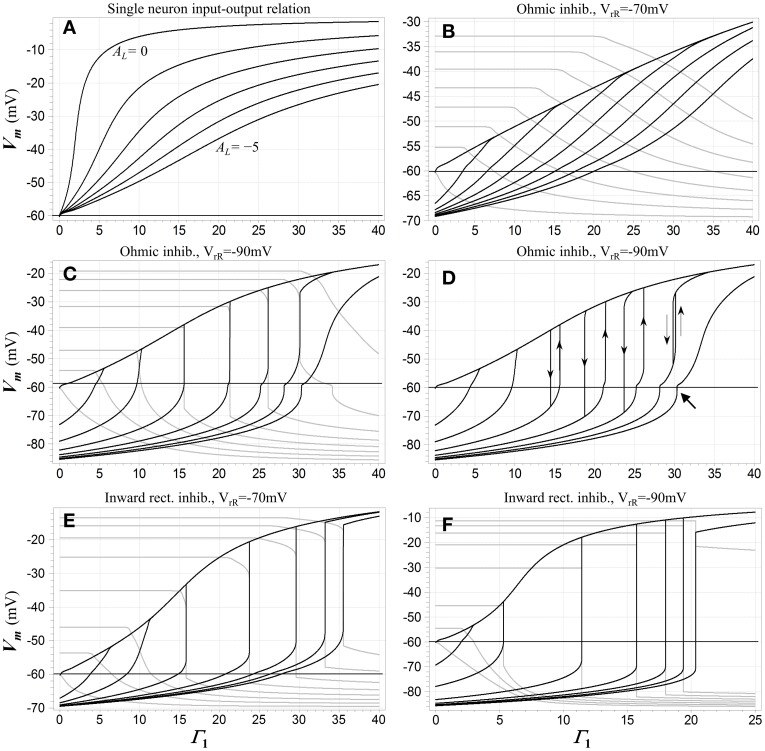
**(A)** A dc input-output characteristic for the network when only a single competitive neuron N_1_ is activated. The inhibitory feedback synapses are ohmic with reversal potential −90 mV. The abscissa is the normalized conductance Γ_1_ of the input (NMDA) synapse onto the neuron, the ordinate is its membrane potential, and the parameter is the loop gain constant *A*_L_, which is varied from 0 to -5 in decrements of −1. **(B-F)** dc transition characteristics for several configurations of the network when two neurons receive excitatory input. The loop gain constant *A*_L_ = −4 in each case. Input Γ_1_ is swept in individual trials, and the parameter is the input Γ_2_ to the second neuron N_2_, which is fixed during each sweep of Γ_1_ but varied from trial to trial. Membrane potential *V*_m 1_ of N_1_ is shown in black and *V*_m 2_ of N_2_ in gray. In **(B-D)**, the inhibitory channels are ohmic and Γ_2_ is stepped from 0 to 35 in increments of 5. In **(B)** the inhibitory reversal potential is −70 mV; in **(C,D)** it is −90 mV. **(C)** shows results for upward sweeps of Γ_1_ while **(D)** shows *V*_m 1_ for sweeps in both directions, which trigger transitions at juxtaposed limit points and illustrate the presence of hysteresis. Directions of transitions are indicated by thin arrows. The heavy arrow at lower right points to a small secondary hysteresis loop (not fully visible at this resolution). In **(E,F)** the inhibitory channels are inward-rectifying, and Γ_1_ is swept upward in both cases. In **(E)** the reversal potential is −70 mV and Γ_2_ is stepped from 0 to 35 in increments of 5; in **(F)** the reversal potential is −90 mV and Γ_2_ is stepped from 0 to 17.5 in increments of 2.5. Resting potential in all cases is −60 mV.

Figure 2 depicts some of these dc characteristics, and illustrates the emergence of WTA behavior. For reference purposes, Figure 2A shows the dc input (expressed as relative NMDAR conductance Γ_1_) vs. output (membrane potential) relationship for a single competitive neuron N_1_ when that neuron is the only one in the network receiving excitatory input. The loop gain constant is the parameter. Membrane potential varies smoothly with input activation, and as might be expected, the slope of this relationship decreases as the strength of inhibitory feedback is increased. Figures 2B–F show the behavior of different configurations of the model when two of the competitive neurons receive excitatory input, and the network transitions from one “winner” to another as the first input exceeds the other. (It should be noted that this transitional behavior applies not just to the two-input case, but to *any* situation in which the two neurons with the largest inputs are the only ones that are able to reach a state of depolarization). When the inhibition is ohmic and mildly hyperpolarizing (Figure 2B), the transitions are gentle and the network cannot reasonably be characterized as winner-takes-all. In the remaining cases, however, there are input ranges for which the transitions are not only sharp but discontinuous. These discontinuities and the hysteretic effects that accompany them are associated with bistable regimes. Such a regime prevails over the range of Γ_1_ values bounded by each hysteresis loop in Figure 2D. In these regions, fixed-point solutions exist in which *either* neuron may be significantly depolarized (i.e., in a “high” state), while the other is either hyperpolarized or depolarized to a lesser extent (in a “low” state). Which of the two solutions might be assumed under quasistatic conditions depends on the history of excitation of the system. The discontinuous jumps depicted in Figures 2C–F represent *limit point* transitions, which occur at *fold bifurcations* that correspond to the boundaries of bistable regimes. Outside of transitions between winners, the state of the winning neuron depends on its input level, and thus carries analog information about that input.

The fixed-point solutions in the model as formulated are shaped by a rich array of nonlinearities, including the NMDAR current-voltage relationship and the neural thresholding function, but also the rectifying characteristics of Kir channels (if present) and saturation of the membrane potential in a “winning” neuron. Numerical analysis shows their structure can be quite complex. Bistability is not observed at all when the inhibition is ohmic and mildly hyperpolarizing, as in Figure 2B. When bistability is possible, it requires a minimum value of the loop gain constant, and when the constant exceeds this minimum, it also requires some minimum level of input activation. This is because bistability is not supported by NMDAR conductance acting in parallel with the resting membrane conductance alone, and there must be sufficient activation of inhibition with characteristics that *do* allow bistability in order to overcome the effects of the resting membrane in a competitive neuron.

Bistable behavior is not observed when only a single neuron received excitatory input. This is due to the dependence of the inhibitory current in such a neuron on its own activation, which is approximately second-order due to feedback. Such a supra-linear characteristic suppresses bistable behavior. However, in the other neurons in the network, this dependence is more nearly *linear*, because they are inhibited below their activation thresholds and do not participate in the feedback. For this reason, when the excitatory input to one of these “losing” neurons is sufficient to bring it to the point of depolarization, bistability may be induced and a limit point crossed, as seen in Figures 2C–F.

Another feature of significance seen in Figure 2 is the fact that the bistable regime with the WTA characteristic—i.e., in which the neurons are in opposing high and low states—ultimately collapses and *vanishes* when the inputs become sufficiently large. This is associated with the fact that at high input levels, saturation of the membrane potential in the winning neuron begins to limit the recruitment of inhibition, ultimately allowing the losing neuron to depolarize and start participating in the feedback process even when its input is no larger than that of the winner. The result is seen in Figures 2C,D, wherein the curves for the largest value of Γ_1_ reflect monostability, and in addition are so gentle in transition that the winner-takes-all characteristic must be regarded as having vanished there as well.

**Figure 3:**
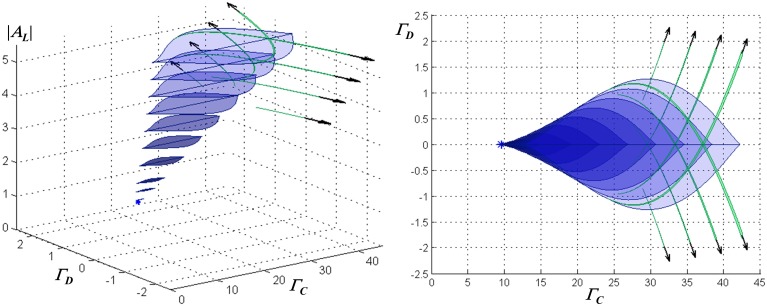
**Characterization of the stationary dynamical regimes of the two-neuron system, for ohmic inhibition with reversal potential −90 mV, in the space spanned by *A*_*L*_, Γ_*C*_ = (Γ_1_ + Γ_2_)/2, and Γ_*D*_ = (Γ_1_ − Γ_2_)/2**. These regimes are symmetric about Γ_*D*_ = 0 due to the physical symmetry of the network. Cross-sections of multistable regimes in Γ_*C*_ − Γ_*D*_ planes are depicted, from an oblique view at left, and in a projection onto the Γ_*C*_ − Γ_*D*_ plane at right. A cusp bifurcation of co-dimension three at [*A*_*L*_ = 1.43, Γ_*C*_ = 9.55, Γ_*D*_ = 0] marks the beginning of multistability and is indicated by a star. Associated with the region that has blue lenticular cross-sections are two sets of stable equilibria, in which one neuron resides in a “low” state (either hyperpolarized or slightly depolarized) and the second in a “high” state (significantly depolarized), or vice-versa. This defines a *WTA bistable regime*. As Γ_*D*_ increases, this region begins and then terminates at co-dimension two cusp bifurcations. Associated with the narrow regions that have green falciform cross-sections are two sets of stable equilibria, in which one neuron resides in one of two low states (one slightly hyperpolarized and the other slightly depolarized) and the second in one of two respective high states. Where a lenticular region intersects a single falciform region, the network is tristable with one neuron residing in one of two low states or a single high state, while the other resides respectively in one of two high states or a single low state. Where all three intersect, the network has four stable states, two high and two low for each neuron. Outside the depicted regions, the network is monostable.

The monostable and WTA bistable regimes described above do not exhaust the dynamical regimes possible in this model. Detailed examination shows the presence in the parameter space of secondary bistable and multistable regimes, which tend to appear as the loop gain and input amplitude are increased. By way of example, Figure 3 shows a portrait of the dynamical structure of the network with instantaneous feedback, for the case of ohmic inhibitory channels and inhibitory reversal potential −90 mV, in the parameter space spanned by the mean or *common-mode* NMDAR activation Γ_*C*_ = (Γ_1_ + Γ_2_)/2, the differential activation Γ_*D*_ = (Γ_1_ − Γ_2_)/2, and *A*_*L*_.

The lenticular regions in Figure 3 show that increasing the loop gain promotes the range of inputs for which bistability is present, as well as the magnitude of hysteresis. The presence of regimes in which two distinct stable “low” states are present (the falciform regions in Figure 3) is related to the neural thresholding function; it may be of academic interest but is of little relevance for the WTA function. (Although not visible at the resolution of the graph, a small hysteresis loop associated with such a regime is present in one of the traces in Figure 2D, and is indicated by a heavy arrow).

When inhibitory conductance is modeled as inward-rectifying, the dc behavior of the two-neuron network is qualitatively similar to the ohmic case in that there is a critical value of the loop gain parameter necessary to support bi- and multi-stable behavior, and above that value a bistable WTA regime appears and then disappears with increasing common-mode NMDAR activation. This bistable behavior is more pronounced: it is supported at higher inhibitory reversal potentials (c.f. Figures 2B,F) and at lower values of the loop gain parameter than when the inhibition is ohmic, and hysteresis is more extensive as well. The co-dimension three cusp bifurcation that marks its beginning occurs at [*A*_*L*_ = 1.09, Γ_*C*_ = 3.81, Γ_*D*_ = 0], which may be compared to the values cited in Figure 3. In addition, the secondary bistable regimes described for the ohmic case—the green falciform regions in Figure 3—are also present.

**Figure 4:**
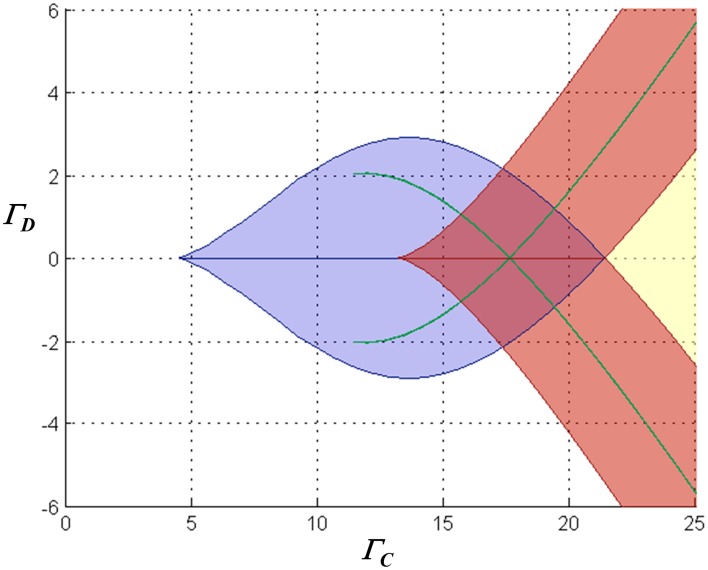
**Characterization of the stationary dynamical regimes of the two-neuron system, for inward-rectifying inhibition with reversal potential −90 mV and *A*_*L*_ = − 4, in the space spanned by Γ_*C*_ = (Γ_1_ + Γ_2_)/2 and Γ_*D*_ = (Γ_1_ − Γ_2_)/2**. The characteristics of the equilibria associated with the blue lenticular region and the green falciform regions are similar to those in the regions with the same color codes in Figure 3. Associated with the chevron-shaped red region are two stable equilibria in which one neuron is in one of two possible high states, and the second respectively in a high or low state. In the yellow region the system is monostable with both neurons in high states (in Figure 2F, the right-hand portion of the traces for largest Γ_2_ are associated with this regime). Where the chevron and the lenticular region alone intersect, the network is tristable with one neuron residing in a high or one of two low states and the other neuron respectively in a low or one of two high states. Where the red, lenticular, and one falciform region intersect, the network has four stable states, and where they all intersect, six stable states.

However, further complexity is introduced by the saturation of inhibitory synaptic current at depolarized membrane potentials. Significantly, as common-mode NMDAR activation increases, mono- and multi-stable regimes emerge in which both neurons can *simultaneously* assume significantly depolarized equilibrium states (a characteristic that precludes a single “winner”). Figure 4 illustrates the various possible dynamical regimes, for one particular choice of the loop gain parameter.

Characterization of the dc behavior of this network has been given for the case in which only a pair of competitive neurons receive non-zero input signals. Simulations show that as the number of active inputs increases, dynamical regimes of increasing complexity become possible. In particular, with inward-rectifying inhibition, multistable states are possible in which three or more competitive neurons are depolarized if their inputs are large enough and similar in magnitude. Nevertheless, with the appropriate loop gain, *there are input ranges for which WTA behavior*—significant depolarization of a single neuron with complete or substantial suppression of activation in all other neurons—*prevails in networks with more than two inputs*, and these ranges subsume multistable regimes in which any one neuron among a set with similar input levels may be a winner. As in the two-neuron case, hysteretic behavior is associated with these regimes and changes in winning states occur at limit points corresponding to regime boundaries.

Finally, it is worth mentioning that evidence such as observations of biphasic inhibitory post-synaptic potentials in various CNS neurons (e.g., Alger and Nicoll, 1979; Davies et al., 1990) suggests that GABA-ergic pathways can activate both GABA_A_ and GABA_B_ receptors in target neurons. If this were to occur in the context of feedback inhibition in this model, it would lead to a mixture of inward-rectifying and non-rectifying inhibitory currents in the competitive neurons. Simulations show that such mixed inhibition results in dc behavior intermediate between the purely rectifying and non-rectifying cases, as might be expected. Interestingly, with a mixture of weakly-hyperpolarizing/ohmic and strongly-hyperpolarizing/inward-rectifying inhibition, a proportion of ohmic conductance as small as 25% of the total can eliminate the multistable solutions with multiple “high” states that are seen with inward-rectifying inhibition alone (e.g., the red regions in Figure 4).

### Dynamic behavior

Under non-stationary operating conditions, time-varying synaptic input signals play the role of driving or forcing functions in the dynamics of the network. Analysis of time-dependent behavior is complicated by the fact that not just neural state, but *dynamical regimes themselves* depend on synaptic inputs, and these regimes can change on a time scale comparable to changes in membrane potential. This can lead to complex time-domain behavior. Nevertheless, a number of conclusions can be reached by time-domain and ac simulations and analysis.

The kinetics of NMDARs associated with the input synapses, which do not play a direct role in the feedback loops, do affect network dynamics in the sense that they have a lowpass filtering effect on the input signals. Conventionally, NMDAR kinetics are regarded as exceptionally slow for an ionotropic receptor (Destexhe et al., 1994; Jahr, 1994; McBain and Mayer, 1994), although they may vary with subtype and with neuromodulatory state (with receptors incorporating the NR2A subunit, for example, having appreciably faster response times (Monyer et al., 1994; Flint et al., 1997; Cull-Candy et al., 2001) than other oligomeric combinations). The response of prototypical slow NMDARs to impulsive inputs (e.g., arrival of a presynaptic action potential or short burst thereof) has a time-to-peak of tens of milliseconds, and decay times on the order of 100 ms or more (Destexhe et al., 1994). Simulation of the ac response of a linearized NMDAR kinetic model (Destexhe et al., 1995) yields a lowpass −3 dB corner frequency at around 1 Hz. One result is that if the presynaptic signaling is spiking in nature, then relative variation in the state of a population of synaptic receptors due to each individual spike is insignificant when the firing frequency is more than a few tens of Herz. The implications of these slow kinetics for network function are that changes in network state may significantly lag the presynaptic input signals that drive them, and also that high-frequency changes in winning states would tend to be suppressed when the network is in a winner-takes-all regime.

An important question with respect to network dynamics is whether the fixed points identified by the stationary analysis are stable or unstable in nature. The answer is determined by the dynamical characteristics of the inhibitory feedback in conjunction with input state and other network parameters. The main focus of this study is on stable WTA behavior and the conditions that support or promote stable regimes in the network, and so loss of stability is considered mainly in this context. However, brief attention is given to operating characteristics in unstable regimes, particularly as pertains to WTA function, although a detailed treatment of unstable dynamics is beyond the scope of the paper.

To examine the issue of fixed-point stability of the feedback circuits, I include dynamic elements in the model network and apply a standard technique of linearization of the governing equations about the fixed points, as detailed in Methods. These elements include membrane capacitance in the competitive neuron, and selectively in the feedback path, a pole to model the effects of membrane conductance and capacitance in the inhibitory interneuron, and cascaded synaptic receptor models.

Consider first the simplest input scenario in which a single competitive neuron is activated. When membrane capacitance is added to the model neuron but the feedback remains instantaneous, a single pole residing in the left half of the complex plane is introduced in the dynamics and the fixed points of the circuit are stable. However, when independent rate equations are also present for one or more states in the feedback path, it is possible for the single-neuron system to lose stability on some subset of its fixed points. With this loss of stability, large-scale limit cycles—i.e., oscillations—occur. Unstable regimes coincide with small, or even negative, incremental membrane conductance in the competitive neuron—a condition that also prevails when a network with multiple active inputs enters a WTA mode of operation. (When the conductance is negative at a fixed point, *instantaneous* feedback *stabilizes* the network, but this effect is compromised with the introduction of sufficient feedback lag.) Conversely, when the system is *not* in a state that would support WTA behavior, it tends to be robustly stable. (This is the case for weakly hyperpolarizing ohmic inhibition, for which consistent fixed-point stability is seen for all feedback dynamics and input conditions considered in the study).

Destabilization of the single-neuron model system can be induced by the addition of even a single feedback pole, if the time constant and the loop gain are large enough. Such instability is enhanced by the introduction of multi-state dynamics to represent synaptic delays. As might be expected, when very slow (metabotropic) GABA_B_ receptors are present, unstable behavior is more pronounced than with the much faster (ionotropic) GABA_A_ receptors, in the sense that it occurs for smaller loop gains and a wider range of inputs. Oscillations in unstable regimes tend to be quite slow for GABA_B_ inhibition (around 4 Hz for the models/parameters used herein), but considerably faster (20–30 Hz) for GABA_A_.

**Figure 5:**
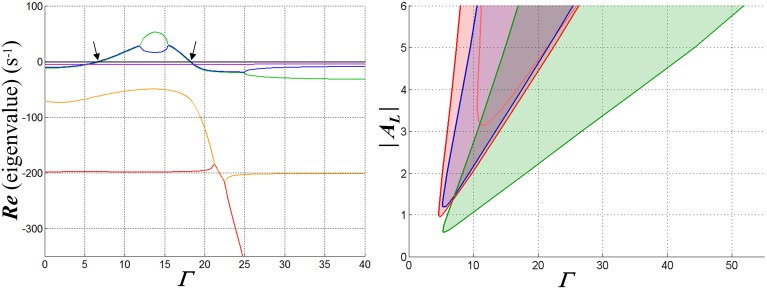
**Graphs illustrating the fixed-point stability characteristics of one configuration of the WTA network when a single input is active**. Inhibitory synaptic conductance is inward-rectifying with reversal potential −70 mV. The resting membrane time constant τ_*R*_ in the competitive neuron is set to 20 ms; membrane charging in the inhibitory interneuron is modeled with a pole with time constant 5 ms, and is cascaded with a four-state model for GABA_B_ receptor dynamics (Destexhe et al., 1995) that introduces three additional poles in the feedback path. At left are depicted the real parts of the poles of the closed-loop system at its fixed points as functions of the input strength Γ, in this case for *A*_*L*_ = −4. Each is coded by a different color. Where two curves overlie, the corresponding poles are complex conjugates. The arrows indicate the points at which a complex pole-pair transitions between left- and right-half-planes and bracket an input range over which the fixed points are unstable. In the middle part of this region, the poles are real and distinct, implying exponential divergence of trajectories away from the fixed point—but large-signal limit cycles (i.e., oscillations) are ultimately assumed by such trajectories. At right, the region over which the network is unstable in the space spanned by Γ and *A*_*L*_ is shown in red (for purposes of comparison, the pink curve shows the boundary of this region when GABA_A_ kinetics are substituted for GABA_B_). In the blue area the membrane conductance in the competitive neuron is negative for the input and inhibitory states at the fixed point, and in the green, WTA bistability would be possible if the input to a second neuron were active (and feedback instantaneous).

**Figure 6:**
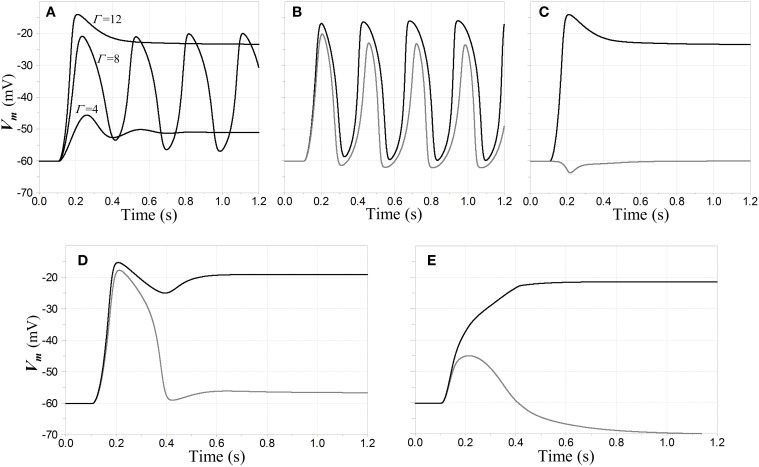
**Oscillatory and stable responses of competitive neurons to step inputs**. **(A–D)** Inhibitory feedback is inward-rectifying with reversal potential −70 mV and *A*_*L*_ = −2; the feedback dynamics include a pole with time constant 5 ms cascaded with a model for GABA_B_ dynamics; and the resting time constant τ_*R*_ = 20 ms in the competitive neuron. The step inputs are convolved with a model for NMDAR kinetics to determine NMDAR conductance values. **(A)** Stability lost and regained with increasing input amplitude, when a single competitive neuron is the only one activated. Membrane potential of the neuron in response to input steps with three different magnitudes of NMDAR conductance Γ is shown. Each step is applied at time *t* = 100 ms. For Γ = 4 the network is in a relatively low-gain stable state; for Γ = 8 it is in an unconditionally unstable state; and for Γ = 12 it is in a stable regime that would support WTA behavior. **(B,C)** Responses when a first neuron N_1_ receives a step input with Γ_1_ = 12 at *t* = 100 ms, and a second neuron N_2_ a step input whose amplitude is 90% that of the first (Γ_2_ = 10.8). Membrane potential of N_1_ is shown in black and that of N_2_ in gray. In **(B)** the two steps are simultaneous, and the network breaks into entrained oscillations, even though it would be stable without the second input. In **(C)** the input to N_2_ is delayed an additional 100 ms, and the network settles to a stable fixed point with a WTA characteristic. In **(D)** the network has the same configuration as in **(A–C)**, except a saturating input-output characteristic has been attributed to the inhibitory interneuron N_FB_. The input steps are synchronous; but although there is a large transient depolarization in N_2_, the entrained oscillations that are present in **(B)** are squelched here. In **(E)** the inhibitory feedback is split between ohmic/weakly-hyperpolarizing and inward-rectifying/strongly-hyperpolarizing, with the former accounting for 25% of the total conductance. The inputs are stepped simultaneously to Γ_1_ = 15, Γ_2_ = 13.5 at *t* = 100 ms.

Analysis shows that when the single-neuron network enters an unstable regime, it does so via a *Hopf bifurcation* (Hopf, 1942; Andronov et al., 1966; Marsden and McCracken, 1976), i.e., the transition of a complex pole-pair between left- and right-half-planes with variation of a parameter—in this case, the input strength. Furthermore, stability is typically lost and then *regained* via such bifurcations as the input increases in magnitude. This characteristic behavior is illustrated in Figure 5, in which stable and unstable regimes are illustrated for a particular network configuration (weakly hyperpolarizing, inward-rectifying inhibition) and choice of feedback dynamics. Qualitatively similar results are obtained for other network configurations that support WTA behavior. Figure 6A shows results of a time-domain simulation in which stability is lost and regained.

Analysis also shows that when stability is lost, it occurs via a *supercritical* Hopf bifurcation (in which a small limit cycle appears around the fixed point and increases in amplitude with increasing input). Stability is also regained via a supercritical Hopf bifurcation in the case of ohmic inhibition, but when the current is inward rectifying it occurs via a *subcritical* Hopf bifurcation (in which the fixed point becomes stable but a large-signal limit cycle remains outside a finite-sized basin of attraction about the fixed point). Thus, sustained oscillatory behavior can persist even after a fixed point has regained stability. The basin of attraction of the fixed point, however, grows rapidly with increasing input levels and eventually subsumes the entire state space.

What are the implications of this analysis for network operation when other inputs are activated? When an unstable regime is present in the single-neuron system, it defines a *minimal range* of input strengths over which the network will be unstable: even if the outputs of other competitive neurons were completely suppressed, sustained oscillations would take place if the largest input were in this range. However, simulations demonstrate that with multiple inputs, *entrained, phase-locked* oscillations can occur when input values are *beyond* this range, but within a regime that supports WTA behavior (e.g., within portions of the green region in Figure 5 that are not overlapped by the red). What would be damped oscillatory behavior in a single neuron can become unstable when additional inputs are activated, even though *the fixed point solutions in this regime are themselves stable* and WTA in character. With sufficient loop gain, this behavior can be seen in any of the model configurations supporting a WTA characteristic. It is associated with the *summation* of competitive outputs onto the inhibitory interneuron: the effect of phase-locked oscillations from the standpoint of an individual neuron is the same as an increase in its inhibitory loop gain, which is naturally destabilizing. This combination of large-signal limit cycles coexisting with fixed point stability is again characteristic of a system that undergoes a subcritical Hopf bifurcation.

The regime in which the network resides depends on the history of activation of the inputs: for example, entrained oscillations may grow when the onset of inputs to two or more competitive neurons is simultaneous, but if the same inputs were staggered in time, the initially-active neuron may settle to a degree that the entire network remains stable after all inputs have become active. Examples of such behavior are shown in the time domain in Figures 6B,C.

**Figure 7:**
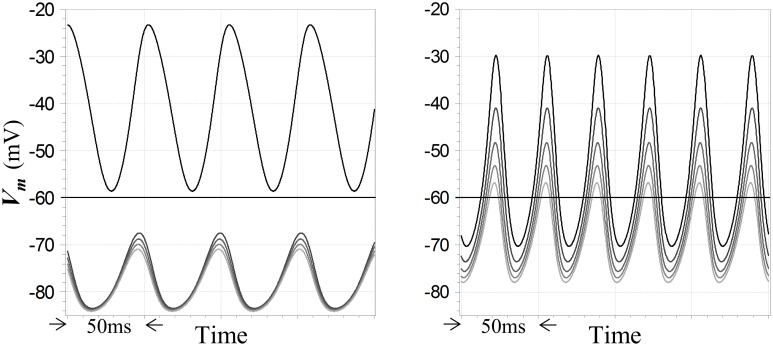
**Winner-takes-all and high gain responses of competitive neurons in unstable regimes**. Five competitive neurons receive step inputs at the start of the simulation; the largest is set to a value leading to instability and the smaller inputs differ by multiples of 5% of the maximum value. Subsequent steady-state oscillations in membrane potentials are depicted. Membrane potentials of the neurons are coded by traces with distinct shades of gray. Feedback dynamics include a pole with time constant 5 ms cascaded with models for GABA_*A*_ receptor dynamics and the resting time constant τ_*R*_ = 20 ms in the competitive neuron. At left, inhibitory feedback is inward-rectifying with reversal potential −90 mV and *A*_*L*_ = −8, and Γ_1_ = 10 for the maximum input; a strong WTA characteristic is seen. At right, inhibition is ohmic with reversal potential −90 mV and *A*_*L*_ = −5, and Γ_1_ = 15 for the maximum input; the response is not strictly WTA in nature but does reflect a high gain. Resting potential in all cases is −60 mV.

What are the implications of oscillatory behavior for the WTA function?—in particular, do peak membrane potentials in unstable regimes show any sort of WTA characteristic, when the corresponding fixed points for those inputs are WTA in nature? This question is germane due to the consistency of the present model with so-called PING models for gamma oscillations in vertebrate cortex (Whittington et al., 2000), along with the finding that such networks can implement an “E%-max winners take all” function (De Almeida et al., 2009)—which has led to the hypothesis that a multiple winners-take-all computation may in fact be a *function* of gamma oscillations (De Almeida et al., 2009). In the present case, the answer is yes: simulations show that WTA behavior can be imputed to oscillatory regimes—but not invariably or strictly. At left in Figure 7 is shown an example of steady-state oscillations in a network of a particular configuration, in which the outputs of the neuron with the largest input exceeds the resting potential throughout its cycle, while the outputs of four other neurons with smaller inputs are uniformly suppressed below the resting potential. Conversely, Figure 7 also shows at right a result for another configuration in which the WTA characteristic is *weakened* by instability, in the sense that membrane potentials in non-winning neurons are not as strongly suppressed as they would be at the corresponding fixed points. However, although all of the neurons in this second case exceed the resting potential during their cycles, the dynamic gain during oscillation is still relatively high, since the smallest of the five inputs is only 20% smaller than the largest. Qualitatively, the behavior in these sorts of oscillatory regimes is seen to depend largely on the difference between the largest and second-largest inputs to the network. More generally, simulations show that unstable behavior can be relatively complex, for example showing evidence of long time-scale modes that mediate a switch between strict WTA behavior and lower-gain excursions as the system evolves in time for particular choices of configuration and input levels. Examination of such complexities are beyond the scope of this study.

Interestingly, unstable behavior in this model may be suppressed by several mechanisms that might plausibly be found in biological neural circuits. One is *saturation* in the inhibitory interneuron, e.g., the limiting of its membrane potential by the finite reversal potential associated with its input synapses—a feature that is perhaps more realistic than the linear model used in the prior analyses. This sort of saturation was investigated in a limited number of simulations, and an example in which it suppresses entrained oscillations is depicted in Figure 6D. A second is a mixture of GABA_A_ and GABA_B_-type inhibition in the competitive neurons, with the faster and more nearly ohmic GABA_A_ channels exerting a stabilizing influence when inward-rectifying channels alone might result in unstable equilibria and limit cycle behavior. In qualitative terms, the fast activation of GABA_A_ channels can allow the network dynamics to remain damped while slower changes in the states of the GABA_B_ channels are ramping up or down—and even though the GABA_A_ channels might not by themselves permit strong WTA behavior, their presence can allow the network to settle into stable WTA states. An example of such behavior is shown in Figure 6E.

Finally, simulations with more general time-varying inputs demonstrate that when the network is in a stable WTA mode (i.e., in the basin of attraction of a stable fixed point), it will subsequently remain stable for any limit-point transitions between winning neurons so long as the largest input remains outside the unconditionally unstable range.

Figure 6 also serves to emphasize graphically the temporal sluggishness of the network response when both slow NMDAR kinetics and GABA_B_ receptor dynamics are present in the model. In order for a network with these dynamics to act as a WTA in a biological context, the time scales on which the input signals operate (i.e., their persistence and the times between changes in their rank ordering) must be on the order of at least several 100 ms. This restriction would seem to preclude the network from functioning as a sequential WTA if its inputs were modulated at frequencies comparable, say, to that of the theta rhythm in mammals. Faster resolution of stable winning and losing states would naturally be the result, however, if faster NMDAR kinetics, and/or mixed GABA_A_ and GABA_B_ inhibition, were present.

## Discussion

The results presented here demonstrate how a neuronal network with NMDA input synapses and lateral inhibition can act as a winner-takes-all circuit, and illuminate some of its significant operating characteristics. The possibility of WTA behavior is conditioned on the characteristics of the inhibitory feedback in the network—in particular, on the amount of loop gain and the electrical characteristics of synaptic ion channels associated with the inhibitory feedback. When WTA behavior is supported, it emerges as the magnitude of inputs to the network increase, with transitions between “winners” taking place with increasing gain, and then becoming abrupt as they are associated with limit points of multistable regimes (stable, that is, if feedback is instantaneous). The output of the winner—quantified herein as its level of membrane depolarization—carries analog information about the magnitude of its input when the network is in a WTA regime. As input strengths increase further, however, such regimes ultimately collapse, giving way again to finite-gain behavior, or to multistable regimes that do not have a WTA characteristic. Thus, if the neuronal network is to operate as a WTA, the maximum NMDAR conductance must be bounded from above by some value that is determined by the strength and the characteristics of the inhibitory feedback. Biophysically, such a bound is a natural constraint that could be imposed by finite numbers of post-synaptic NMDA receptors available, and/or limits on presynaptic neurotransmitter release, at the inputs to the competitive neurons.

In a multistable regime in which transitions between winners are abrupt, if one neuron is established as a winner then the input to another neuron must exceed that of the first by some finite amount in order for such a transition to occur. This means that at any instant in time the “winning” neuron may not actually be the one with the largest input. Such hysteretic behavior, however, can serve the purpose of preventing transitions due to noise or to relatively small differences between the input levels of two contending neurons—in other words, it can prevent “jitter” between winners and could be interpreted as requiring some level of *confidence* in the result before a transition can occur.

Analysis of time-domain behavior of the network with plausible dynamics in the inhibitory feedback loops show that it is possible for it to destabilize. With sufficient loop gain, as one or more inputs increase in magnitude, the network can enter a regime in which its fixed-point states (including some that would be multistable with instantaneous feedback) are unstable. When it receives sustained inputs in this regime, the network evolves into large-signal oscillations (*entrained* oscillations when there is more than one input). The peak membrane potentials during such oscillations may in some circumstances be regarded as WTA in character, and generally they reflect a high dynamic gain although the WTA function may be compromised relative to the corresponding fixed point solutions. As inputs increase further, the system exits this regime and enters a second in which there are stable fixed points with WTA characteristics—but these may coexist with large-signal limit cycles.

Destabilization of the network is most pronounced (i.e., occurs at lower loop gains and covers a larger range of input strengths) when the slow dynamics of (second-messenger) GABA_B_ receptors are associated with the inhibitory synapses in the feedback loops. Very small or negative membrane conductance values in the competitive neurons, which are more prevalent when the inhibitory current is modeled as inward-rectifying, also promote instability. Thus, it might be expected that lateral inhibition implemented with GABA_B_ synapses would compromise the ability of such a network to implement a stable WTA function. However, other biologically-plausible characteristics can mitigate against unstable behavior. When the simplifying assumption of linearity in the inhibitory interneuron is relaxed and saturating effects introduced, it can squelch large-signal oscillations and establish a stable WTA regime (although the network may pass through an unconditionally unstable regime at lower input values to reach it). In addition, the presence of GABA_A_ as well as GABA_B_ receptors in the inhibitory feedback pathway can have a significant stabilizing effect.

Although tangential to the main subject of the paper, it is of interest to note that when this model is in unstable regimes, it produces spontaneous oscillations whose frequencies are similar to the theta rhythm when GABA_B_ kinetics are present in the feedback, and when GABA_A_ kinetics are present, to the gamma rhythm (although somewhat slower for the particular parameter values used). It is well-known that the gamma as well as beta1 and beta2 bands are dependent on the time constants of GABA_A_ receptor kinetics (Whittington et al., 2000). The results herein confirm that gamma-like oscillations can arise in a PING architecture with fixed or slowly-varying inputs, and in addition that behavior in these regimes is generally consistent with the hypothesis that a (single or multiple) winners-take-all function might be an inherent feature of gamma oscillations (De Almeida et al., 2009).

At this point, a word on the magnitudes of NMDAR conductance and the inhibitory loop gain constant in the model is in order. As defined in Methods, the loop gain parameter is simply the gain around any one of the feedback loops in the network when it is at rest; conceptually, this means that if a feedback loop in the quiescent network were broken at the level of a competitive neuron, then an increment of presynaptic depolarization at its output synapse would come back around the loop to effect a hyperpolarization |*A*_*L*_| times as large in the cell itself. The synaptic weightings implied by the single-digit values of *A*_*L*_ considered in the study would therefore seem to be quite plausible. Analysis also suggests that the densities of active NMDARs required to achieve the reported network behaviors are biologically plausible. The numerical values assumed by the NMDAR conductance parameter may be misleading in this regard, because by convention it refers to the slope conductance at the channel *reversal potential*, where magnesium blockade is minimal. Where the blockade is more pronounced—as over most of the operating range of the network—the slope conductance of the NMDAR channels (relative to resting conductance) is typically much smaller than the Γ parameter itself.

In summary, if such WTA microcircuits are in fact implemented in biological neuronal networks, they would endow nervous system with a building block of appreciable computation power (Maass, 2000), and could support functions ranging from the abstract (e.g., function approximation) to the psychophysically- or ethologically-grounded (e.g., selective attention). But although the functional implications at the level of the primitive circuit are clear, any possible role in broader contexts (e.g., of the cortical circuits documented in the extensive literature on recurrent/reverberating networks) requires further consideration. (For example, observations of an “inverted U-shaped” dependence of persistent activity on external drive in assemblies of cortical cells (Williams and Goldman-Rakic, 1995; Brunel and Wang, 2001) could be related to the behavior of this circuit as it is driven up to, through, and then past a WTA regime by increasing input strength.) Finally, although the subject of *learning* (e.g., by long-term potentiation) is beyond the scope of this paper, it is clear that the model represents a biophysically-plausible component of a substrate for competitive learning. It would provide for Hebbian modification exclusive to a single winning neuron during any cycle of activation, and strengthening of excitatory synapses within the network would predispose neurons so affected to future wins.

## Methods

### Mathematical model

In order to establish the fundamental behavior of the network, I consider a model which is intended to be as simple as practical while maintaining enough biological fidelity to characterize the possible range of behaviors of the neuronal networks it represents. The following specifications describe the framework for this model:

- All neurons in the network shown in Figure 1 are electrically compact and representable as single electrical compartments;
- Each neuron has a passive membrane conductance that can be approximated as ohmic, with an associated resting membrane potential (set to −60 mV in simulations), and these characteristics remain fixed in time;
- The competitive neurons are biophysically homogeneous, i.e., they have the same resting membrane properties, and the synapses of each class are weighted identically from neuron to neuron;
- Synapses onto the competitive neurons are represented as variable membrane conductances in the postsynaptic cell with values determined by presynaptic membrane potential. The nonlinear voltage dependence of NMDAR channel current is based on the model and parameters of Jahr and Stevens (1990). The reversal potential for the inhibitory synapses is assigned a value of either −70 mV or −90 mV in simulations, and inward-rectifying channels are modeled with the *ad hoc* current-voltage relationship used by Shoemaker (2011);
- Neuronal activation involves a thresholding operation corresponding to half-wave rectification: output synapses are activated in proportion to depolarization but are inactive during hyperpolarization;
- For the sake of simplicity, the inhibitory interneuron is approximated with a linear model unless otherwise indicated. It performs a simple summation of its inputs, and its behavior under non-stationary conditions is represented with first-order linear dynamics;
- Postsynaptic conductance under non-stationary conditions is determined by convolution of the presynaptic activation with an impulse response function representing a linearized model of receptor kinetics (as given in Destexhe et al., 1995 for NMDARs, GABA_B_, and GABA_A_ receptors).

This simple model neglects the generation of action potentials, and is thus applicable to cases in which the neurons either operate with graded potentials, or for spiking cells, in which outputs can be characterized by a spike rate that may in turn be related to mean membrane depolarization. In addition, although spiking input pathways could be represented with impulsive inputs in the time domain, continuous-time inputs are used throughout. This may be justified by the lowpass-filtering effects of slow NMDAR kinetics at the input synapses, as discussed in Section Dynamic Behavior. The model is not intended to represent networks in which spike timing is in some way critical, or in which individual spikes are otherwise significant computationally.

In any of the competitive neurons N_1_, N_2_, N_3_, …, the membrane current balance equation may be written in the form

(1) $$\begin{array}{l} G_{N}f_{N}(V_{m})+G_{I}f_{I}(V_{m};V_{rI})+G_{R}f_{R}(V_{m};V_{rR})\\ \;+C_{m}dV_{m}/dt=0, \end{array}$$

where *V*_*m*_ is membrane potential, and *G*_*N*_, *G*_*I*_, and *G*_*R*_ are conductance parameters and the functions *f*_*N*_(*V*), *f*_*I*_ (*V;V*_*r*_), and *f*_*R*_ (*V;V*_*r*_) characterize the voltage dependence of channel currents for NMDARs, inhibitory receptors, and resting membrane conductance, respectively. The parameter *V*_*r*_ in this notation indicates reversal potential; *V*_*rI*_ and *V*_*rR*_ are reversal potentials for inhibitory ion channels and resting conductance, respectively. The reversal potential for NMDAR channels is assumed to be 0V and is not included as a parameter. After the convention in (Shoemaker, 2011), the functions *f*_*N*_, *f*_*I*_, and *f*_*R*_ are defined with dimensions of volts and are normalized to unity slope at their reversal potentials. *G*_*N*_ is assumed to be governed by the activation of the NMDA synapse associated with the input to the neuron, and *G*_*I*_ by the activation of the inhibitory feedback synapse. *C*_*m*_ is membrane capacitance. Assuming that all competitive units in the network (i.e., neurons and synapses of each class that contact them) are biophysically identical, I append a numerical index to quantities that may vary between them, i.e., *V*_*m i*_ and *G*_*Ni*_, *i* = 1…*n*, to indicate that they apply to the *i*th neuron N*_*i*_* among a set of n. In addition, it is convenient to eliminate a free parameter by multiplying (1) by *R*_*R*_ = 1/G_*R*_, yielding the set of equations

(2) $$\begin{array}{l} \Gamma_{i}f_{N}(V_{m\;i})+\Gamma_{I}f_{I}(V_{m\;i};V_{rI})\;\\ \;+f_{R}(V_{m\;i};V_{rR})+\tau_{R}dV_{m\;i}/dt=0,\;i=1\ldots n, \end{array}$$

where Γ_*i*_ = *R*_*R*_ *G*_*n i*_ and Γ_*I*_ = *R*_*R*_ *G*_*I*_ are dimensionless synaptic conductances expressed relative to the resting conductance, and τ_*R*_ = *R*_*R*_ *C*_*m*_ is the resting membrane time constant. The normalized dc transmembrane current *I*_*mi*_ for the *i*th neuron is

(3) $$\begin{array}{l} I_{mi}=\Gamma_{i}f_{N}(V_{m\;i})+\Gamma_{I}f_{I}(V_{m\;i};V_{rI})\\ \;+f_{R}(V_{m\;i};V_{rR}),\;i=1\ldots n, \end{array}$$

and collectively, the zeros of these currents define the fixed points of the system.

**Figure 8:**
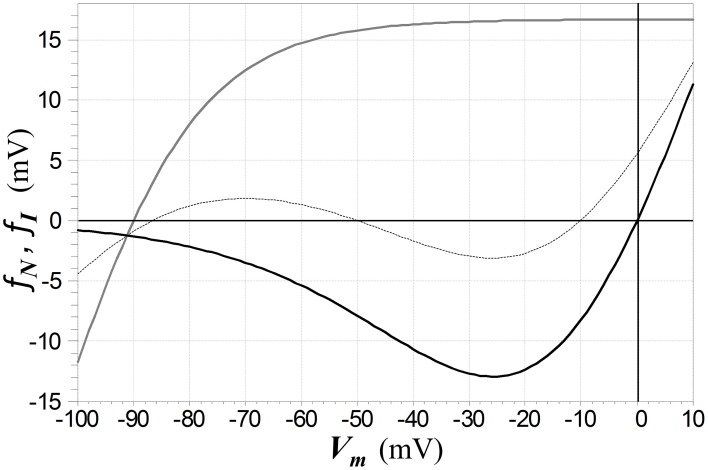
**Functions *f*_*N*_ (solid black) and *f*_*I*_ (solid gray) that respectively characterize the voltage-dependence of NMDAR ion channels and Kir channels (as defined by the second line in 5)**. The Kir channel reversal potential is set to −90 mV in this example. The dashed curve depicts the current-voltage relation of a membrane (with current normalized by a unit conductance to give a voltage) in which channels of both types are active, with the NMDAR conductance twice than of the Kir conductance, leading to electrical bistability. The middle zero of this function represents an unstable equilibrium and the other two, stable equilibria.

The voltage dependence for NMDAR channel current is based on the model and parameters of Jahr and Stevens (1990), and the corresponding function *f*_*N*_ may be written

(4) $$f_{N}(V_{m})=\frac{(1+b)V_{m}}{1+b\exp(-kV_{m})\;},$$

where *b* = 0.28 mM^−1^ · [Mg^2+^] = 0.336 under an assumed extracellular magnesium concentration [Mg^2+^] = 1.2 mM, and where *k* = 62V^−1^. The voltage dependence of inhibitory synaptic ion channels takes one of the two forms

(5) $$\begin{array}{l} f_{I}(V_{m};V_{rI})=\;V_{m}-V_{rI}\;(\text{ohmic channels})\\ f_{I}(V_{m};V_{rI})=\frac{d\{\tanh[(V_{m}-V_{rI}-c)/d]-e\}}{1-\tanh^{2}(c/d)}\;(\text{Kir channels}) \end{array},$$

where the form for Kir channels is the *ad hoc* function used in Shoemaker (2011), with *d* = 25 mV, *e* = 0.5, and *c* = −13.73 mV (which places the zero-crossing of the function at *V*_*rI*_), and where tanh denotes hyperbolic tangent. The voltage-dependent functions for NDMAR and Kir channels are depicted in Figure 8.

Under the assumption that the resting conductance is ohmic,

(6) $$f_{R}(V_{m};V_{rR})=\;V_{m}-V_{rR}.$$

The output of each competitive neuron is computed by applying a thresholding function *h*(*V*_*m*_) ≅ max[(*V*_*m*_ − *V*_*rR*_), 0] to the membrane potential to obtain the depolarization of the neuron above the resting potential. In practice, the function on the right-hand side of this expression is splined with a quadratic function over the range *V*_*rR*_ ± 1 mV to maintain continuity of both the thresholding function and its derivative. The output is identically zero for *V*_*m*_ < *V*_*rR*_ − 1 mV.

I assume that the relationship between the NMDAR conductance Γ_*i*_ and the input *IN*_*i*_ to neuron N*_i_* is linear, and thus without loss of generality equate the two for purposes of stationary analysis. For dynamics, I assume that Γ_*i*_ = *h*_N_^*^ *IN*_*i*_, where ^*^ indicates temporal convolution, *h*_*N*_ is a dimensionless unit impulse function based on the linearized 4-state NMDAR kinetic model of Destexhe et al. (1995), and *IN*_*i*_ assumes the dimensions and scaling of the NMDAR conductance.

The inhibitory conductance Γ_*I*_ that is driven by the interneuron N_*FB*_ takes on a common value for all competitive neurons, but in general is variable and dependent on the states of those units via a feedback equation. With the assumptions of electrical compactness and approximate linearity in N_FB_, under stationary conditions it may be written in the form

(7) $$\Gamma_{I}=K\cdot \sum_{j=1}^{n}h(V_{mj}-V_{rR}),$$

where *K* is a constant of proportionality that includes the effects of the strength or weight of the input and output synapses of N_FB_.

Now consider a single-neuron system with ohmic inhibitory conductance and with no input activation. Conceptually, break the feedback loop at the presynaptic side of the input to N_FB_. For an ideal thresholding function (i.e., ignoring the spline used in the numerical simulations), the right-handed derivative of Γ_*I*_ with respect to the presynaptic membrane potential *V*_*m Pre*_ is simply *d*Γ^+^_*I*_/*dV*_*m Pre*_ = *K* for any *V*_*m Pre*_ ≥ *V*_*rR*_. The derivative of the membrane potential of the competitive neuron with respect to Γ_*I*_ is found to be *dV*_*m*_/*d*Γ_*I*_ = (*V*_*rI*_ − *V*_*m*_)/(Γ_*I*_ + 1) by implicit differentiation, and evaluating this in the state that the closed-loop system assumes when it is not excited, i.e., *V*_*m*_ = *V*_*rR*_ and Γ_*I*_ = 0, gives *dV*_*m*_/*d*Γ_*I*_ = (*V*_*rI*_ − *V*_*rR*_). Thus, the loop gain under these conditions is

(8) $$\frac{dV_{m}}{dV_{m\;Pre}}=\frac{d\Gamma_{I}^{+}}{dV_{m\;Pre}}\cdot \frac{dV_{m}}{d\Gamma_{I}}=K(V_{rI}-V_{rR}).$$

I take the rightmost expression in (8) as the *loop gain parameter*:

(9) $$A_{L}\equiv K(V_{rI}-V_{rR}).$$

When the inhibitory channels are inward-rectifying, the quiescent loop gain is not precisely equal to this expression due to the nonlinearity of their channel current-voltage relation, but I maintain the same definition of *A*_*L*_ so that it bears the same relation to the synaptic weights in the feedback paths. This allows straightforward comparison of the behavior of the two different types of inhibitory channels considered.

With this definition, (7) becomes

(10) $$\Gamma_{I}=\frac{A_{L}\cdot \sum_{j}h(V_{mj}-V_{rR})}{\left(V_{rI}-V_{rL}\right)},\;j=1\ldots n.$$

When considering the dynamic behavior introduced into the feedback loop by the kinetics of the inhibitory channels, the expression in (10) is replaced by

(11) $$\Gamma_{I}=h_{I}*\frac{A_{L}\cdot \sum_{j}h(V_{mj}-V_{rR})}{\left(V_{rI}-V_{rL}\right)},\;j=1\ldots n,$$

where *h*_*I*_ is a dimensionless unit impulse function based on one of the linearized four-state GABA kinetic models of (Destexhe et al., 1995).

Finally, saturation of membrane potential in the inhibitory interneuron, when present, is modeled via the trans-synaptic response at its inputs. Identify (without loss of generality, since any scaling may be absorbed in the loop gain constant) the net input to N_FB_, $\sum_{j=1}^{n}h$(*V*_*m j*_ − *V*_*rR*_), with a current that is expressed across a unit membrane resistance to determine the depolarization Δ*V*_*mFB*_. When saturation is present, Δ*V*_*mFB*_ is determined implicitly by the equation Δ*V*_*mFB*_ = $\sum_{j=1}^{n}h$(*V*_*m j*_ − *V*_*rR*_) · (Δ*V*_*mFB*_ + *k*_*sat*_ · *V*_*rR*_)/(*k*_*sat*_ · *V*_*rR*_), which gives a hyperbolic input/output dependence as would be imposed by a limited reversal potential associated with the input synapses to N_*FB*_. The value *k*_*sat*_ = 2 was used in simulations; with this value, full depolarization of a single competitive neuron (i.e., to a membrane potential of zero) drives the inhibitory interneuron halfway to saturation.

Stability analysis is carried out via linearized first-order state equations, written in terms of deviations of the states (indicated below by the prefix Δ) from their fixed-point values. The membrane potential *V*_*m*_ of the single active competitive neuron is one state. The kinetics of the input synapses to the interneuron N_FB_ are neglected, and the first state *V*_*FB*_ in the feedback loop is assumed proportional to the membrane depolarization of N_FB_. (In practice, the scaling of the entire feedback loop is absorbed into this state.) The dynamics due to membrane resistance and capacitance contribute a single pole with time constant τ_*FB*_. When included, the dynamics of the output synapses, as represented by one or the other of the GABA receptor models of (Destexhe et al., 1995), are cascaded with this pole. These models include two “closed” states, *C*_1_ and *C*_2_, where *C*_1_ combines with a ligand *L* and may then transition to *C*_2_, from which an inactive state *D* and an open (conductive channel) state *O* are accessible. I associate *C*_1_ + *L* with *V*_*FB*_, leaving three independent states that generate three additional poles. The state *O* is associated with the inhibitory feedback conductance Γ_*I*_ in the target competitive neuron (the weight of the inhibitory synapse being, without loss of generality, absorbed into the loop gain parameter).

With these conventions, the linearized dynamic equations for the closed-loop system with feedback synapse dynamics can be written in vector-matrix form:

(12) $$\left|\begin{array}{c} {\dot{V}}_{m}\\ {\dot{V}}_{FB}\\ {\dot{C}}_{2}\\ \dot{D}\\ {\dot{\Gamma }}_{I} \end{array}\right|=\left|\begin{array}{ccccc} \frac{-1}{\tau_{R}}\frac{dI_{m}}{dV_{m}} & 0 & 0 & 0 & \frac{-1}{\tau_{R}}\frac{dI_{m}}{d\Gamma_{I}}\\ \frac{1}{\tau_{FB}}\frac{A_{L}}{\left(V_{rR}-V_{rI}\right)} & \frac{-1}{\tau_{FB}} & 0 & 0 & 0\\ 0 & k_{FB} & -r_{\Sigma } & r_{5} & r_{4}\\ 0 & 0 & r_{6} & -r_{5} & 0\\ 0 & 0 & r_{3} & 0 & -r_{4} \end{array}\right|\left|\begin{array}{c} \Delta V_{m}\\ \Delta V_{FB}\\ \Delta C_{2}\\ \Delta D\\ \Delta \Gamma_{I} \end{array}\right|,$$

where *I*_*m*_ is the normalized dc transmembrane current in the competitive neuron as given in (3), *k*_*FB*_ is a scaling constant yielding a unit step response from the receptor dynamics, and the partial derivatives are evaluated at the fixed point under consideration. Rate constants *r*_2_ through *r*_6_ are as defined by Destexhe et al. (1995) for the receptor type under consideration, with *r*_Σ_ ≡ *r*_2_ + *r*_3_ + *r*_6_. The eigenvalues of the matrix on the right-hand side of (11) correspond to the poles of the system.

### Numerical analysis

Simulations were performed primarily with SPICE (Simulation Program with Integrated Circuit Emphasis), a tool that is intended for electronic circuit simulations and is optimized for solution of stiff nonlinear equations. Discrete elements are described by electrical constitutive relations involving their interconnection points or nodes, and governing equations for interconnected circuits are automatically generated by application of Kirchhoff's current law (conservation of charge). Built-in linear elements or user-definable nonlinear voltage-controlled current sources were used for the implementation of nonlinear membrane conductances. SPICE can solve static equations to obtain dc/equilibrium states, and dc sweeps of inputs were performed to determine input/output relations (with sweeps in both directions used to identify limit point transitions). Membrane current-voltage relations could be tested by sweeping a virtual “voltage clamp” with the input and inhibitory conductances held at fixed-point values. SPICE also allows time-domain or transient simulations of dynamical systems, and was used to compute outputs in response to various input scenarios (such as the step functions in Section Dynamic Behavior). Analogical circuits were constructed to implement receptor dynamic models. The product T-SPICE (Tanner Research, Monrovia, CA) was used for the study.

In addition, MATLAB (MathWorks, Natick, MA) was used to compute the poles of the system in the stability analysis. MATLAB and Excel (Microsoft, Redmond, WA) were used for supporting analysis and plot generation.

### Conflict of interest statement

The author declares that the research was conducted in the absence of any commercial or financial relationships that could be construed as a potential conflict of interest.

## References

[B1] Alger B. E. Nicoll R. A. (1979). GABA-mediated biphasic inhibitory responses in hippocampus. Nature 281, 315–317. 10.1038/281315a0 551280

[B2] Amari S.-I. (1972). Characteristics of random nets of analog neuron-like elements. IEEE Trans. Syst. Man Cybern. SMC- 2, 643–657.

[B3] Andreou A. G. Boahen K. Pavasovic A. Jenkins R. E. Strohbehn K. (1991). Current mode subthreshold MOS circuits for analog VLSI neural systems. IEEE Trans. Neural Netw. 2, 205–213. 10.1109/72.80331 18276373

[B4] Andronov A. A. Vitt A. A. Khaikin S. E. (1966). Theory of Oscillators, eds Immirzi F. (Translator) and W. Fishwick. Oxford: Pergamon Press.

[B5] Baishnab K. L. Nag A. Talukdar F. A. (2010). A novel high precision low power current mode CMOS winner-take-all circuit. Int. J. Eng. Sci. Technol. 2, 1384–1390.

[B6] Brunel N. Wang X. (2001). Effects of neuromodulation in a cortical network model of object working memory dominated by recurrent inhibition. J. Comput. Neurosci. 11, 63–85. 10.1023/A:1011204814320 11524578

[B7] Chen Y. McKinstry J. L. Edelman G. M. (2013). Versatile networks of simulated spiking neurons displaying winner-take-all behavior. Front. Comput. Neurosci. 7:16. 10.3389/fncom.2013.00016 23515493 PMC3601301

[B8] Coultrip R. Granger R. Lynch G. (1992). A cortical model of winner-take-all competition via lateral inhibition. Neural Netw. 5, 47–54 10.1016/S0893-6080(05)80006-1

[B9] Cull-Candy S. Brickley S. Farrant M. (2001). NMDA receptor subunits: diversity, development and disease. Curr. Opin. Neurobiol. 11, 327–335. 10.1016/S0959-4388(00)00215-4 11399431

[B10] Davies C. H. Davies S. N. Collingridge G. L. (1990). Paired-pulse depression of monosynaptic GABA-mediated inhibitory postsynaptic responses in rat hippocampus. J. Physiol. 424, 513–531. 10.1113/jphysiol.1990.sp018080 2167975 PMC1189826

[B11] De Almeida L. Idiart M. Lisman J. E. (2009). A second function of gamma frequency oscillations: an E%-max winner-take-all mechanism selects which cells fire. J. Neurosci. 29, 7497–7503. 10.1523/JNEUROSCI.6044-08.2009 19515917 PMC2758634

[B12] Destexhe A. Mainen Z. F. Sejnowski T. (1995). Fast kinetic models for simulating AMPA, NMDA, GABAA and GABAB receptors, in The Neurobiology of Computation, ed Bower J. (Norwell, MA: Kluwer Academic Press), 9–14.

[B13] Destexhe A. Mainen Z. Sejnowski T. J. (1994). Synthesis of models for excitable membranes, synaptic transmission, and neuromodulation using a common kinetic formalism. J. Comput. Neurosci. 1, 195–230. 10.1007/BF00961734 8792231

[B14] Deweerth S. P. Morris T. G. (1995). CMOS current mode winner-take-all circuit with distributed hysteresis. Electron. Lett. 31, 1051–1053 10.1049/el:19950729

[B15] Ermentrout B. (1992). Complex dynamics in winner-take-all neural nets with slow inhibition. Neural Netw. 5, 415–431 10.1016/0893-6080(92)90004-3

[B16] Fish A. Yadid-Pecht O. (2001). CMOS current/voltage mode winner-take-all circuit with spatial filtering, in 2001 IEEE International Symposium on Circuits and Systems (Sydney, NSW), 636–639.

[B17] Flint A. C. Maisch U. S. Weishaupt J. H. Kriegstein A. R. Monyer H. (1997). NR2A subunit expression shortens NMDA receptor synaptic currents in developing neocortex. J. Neurosci. 17, 2469–2476. 9065507 10.1523/JNEUROSCI.17-07-02469.1997 PMC6573498

[B18] Fowler C. E. Aryal P. Suen K. F. Slesinger P. A. (2007). Evidence for association of GABA(B) receptors with Kir3 channels and regulators of G protein signalling (RGS4) proteins. J. Physiol. 580(Pt 1), 51–65 10.1113/jphysiol.2006.123216 17185339 PMC2075413

[B19] Grossberg S. (1973). Countour enhancement, short term memory, and constancies in reverberating networks. Stud. Appl. Math. 52, 213–257.

[B20] Handrich S. Herzog A. Wolf A. Herrmann C. S. (2009). A biologically plausible winner-takes-all architecture, in Emerging Intelligent Computing Technology and Applications, With Aspects of Artificial Intelligence. Lecture Notes in Computer Science Vol. 5755, eds Huang D.-S. Jo K.-H. Lee H.-H. Kang H.-J. Bevilacqua V. (Berlin Heidelberg: Springer), 315–326.

[B21] Hopf E. (1942). Abzweigung einer periodischen Lösung von einer stationären Lösung eines Differentialsystems. Ber. der Math. Klasse der Sächs. Akad. der Wiss. Leipzig 94, 1–22.

[B22] Indiveri G. (2001). A current-mode hysteretic winner-take-all network, with excitatory and inhibitory coupling. Analog Integr. Circuits Signal Process. 28, 279–291 10.1023/A:1011208127849

[B23] Jahr C. (1994). NMDA receptor kinetics and synaptic function. Semin. Neurosci. 6, 81–86 10.1006/smns.1994.1011

[B24] Jahr C. Stevens C. F. (1990). Voltage dependence of NMDA-activated macroscopic conductances predicted by single-channel kinetics. J. Neurosci. 10, 3178–3182. 1697902 10.1523/JNEUROSCI.10-09-03178.1990 PMC6570236

[B25] Johnston G. A. R. (1996). GABAA receptor pharmacology. Pharmacol. Ther. 69, 173–198. 10.1016/0163-7258(95)02043-8 8783370

[B26] Kaupmann K. Schuler V. Mosbacher J. Bischoff S. Heid J. Froestl W.. (1998). Human gamma-aminobutyric acid type B receptors are differentially expressed and regulate inwardly rectifying K+ channels. Proc. Natl. Acad. Sci. U.S.A. 95, 14991–14996. 10.1073/pnas.95.25.14991 9844003 PMC24563

[B27] Koch C. Ullman S. (1985). Shifts in selective visual attention: toward the underlying neural circuitry. Hum. Neurobiol. 4, 219–227. 3836989

[B28] Lau K. T. Lee S. T. (1998). A CMOS winner-takes-all circuit for self-organizing neural networks. Int. J. Electron. 84, 131–136. 10.1080/00207219813489620421180

[B29] Lazarewicz M. T. Ang C.-W. Carlson G. C. Coulter D. A. Finkel L. H. (2006). Analysis of NMDA-dependent voltage bistability in thin dendritic compartments. Neurocomputing 69, 1025–1029 10.1016/j.neucom.2005.12.038

[B30] Lazzaro J. Ryckebusch S. Mahowald M. A. Mead C. A. (1989). Winner-take-all networks of O(N) complexity, in Advances in Neural Information Processing Systems 1, ed Touretzky D. S. (San Francisco, CA: Morgan Kaufmann), 703–711.

[B31] Maass W. (2000). On the computational power of winner-take-all. Neural Comput. 12, 2519–2535. 10.1162/089976600300014827 11110125

[B32] Mao Z.-H. Massaquoi S. G. (2007). Dynamics of winner-take-all competition in recurrent neural networks with lateral inhibition. IEEE Trans. Neural Netw. 18, 55–69. 10.1109/TNN.2006.883724 17278461

[B33] Marsden J. E. McCracken M. (1976). The Hopf Bifurcation and Its Applications. New York, NY: Springer-Verlag.

[B34] McBain C. J. Mayer L. (1994). N -methyl-D-aspartic receptor structure and function. Physiol. Rev. 74, 723–760. 8036251 10.1152/physrev.1994.74.3.723

[B35] Monyer H. Burnashev N. Laurie D. J. Sakmann B. Seeburg P. H. (1994). Developmental and regional expression in the rat brain and functional properties of four NMDA receptors. Neuron 12, 529–540. 10.1016/0896-6273(94)90210-0 7512349

[B36] Mott D. D. Lewis D. V. (1994). The pharmacology and function of central GABA receptors, in International Review of Neurobiology, eds Bradley R. J. Harris R. A. (Amsterdam: Elsevier), 97–224. 10.1016/s0074-7742(08)60304-9 7822122

[B37] Nowak L. Bregestovski P. Ascher P. Herbert A. Prochiantz A. (1984). Magnesium gates glutamate-activated channels in mouse central neurons. Nature 307, 462–465. 10.1038/307462a0 6320006

[B38] Olsen R. W. DeLorey T. M. (1999). Ch. 16: GABA and Glycine, in Basic Neurochemistry: Molecular, Cellular, and Medical Aspects, eds Siegel G. J. Agranoff B. W. Fisher S. K. Albers R. W. Uhler M. D. (Philadelphia: Lippincott-Raven).

[B39] Rumelhart D. Zipser D. (1987). Feature discovery by competitive learning, in Parallel Distributed Processing, eds Rumelhart D. E. Zipser D. (Cambridge, MA: MIT Press), 151–193.

[B40] Sanders H. Berends M. Major G. Goldman M. S. Lisman J. E. (2013). NMDA and GABAB (KIR) conductances: the “perfect couple” for bistability. J. Neurosci. 33, 424–4299. 10.1523/JNEUROSCI.1854-12.2013 23303922 PMC3572916

[B41] Shoemaker P. A. (2011). Neural bistability and amplification mediated by NMDA receptors: analysis of stationary equations. Neurocomputing 74, 3058–3071 10.1016/j.neucom.2011.04.018

[B42] Sodickson D. L. Bean B. P. (1996). GABAB receptor-activated inwardly rectifying potassium current in dissociated hippocampal CA3 neurons. J. Neurosci. 16, 6374–6385. 8815916 10.1523/JNEUROSCI.16-20-06374.1996 PMC6578909

[B43] Whittington M. Traub R. Kopell N. Ermentrout B. Buhl E. (2000). Inhibition-based rhythms: experimental and mathematical observations on network dynamics. Int. J. Psychophysiol. 38, 315–336. 10.1016/S0167-8760(00)00173-2 11102670

[B44] Williams G. V. Goldman-Rakic P. S. (1995). Modulation of memory fields by dopamine D1 receptors in prefrontal cortex. Nature 376, 572–575. 10.1038/376572a0 7637804

[B45] Winder S. A. (1999). A model for biological winner-take-all neural competition employing inhibitory modulation of NMDA-mediated excitatory gain. Neurocomputing 26–27, 587–592 10.1016/S0925-2312(98)00167-2

[B46] Yuille A. L. Geiger D. (2003). Winner-take-all networks, in The Handbook of Brain Theory and Neural Networks, ed Arbib M. A. (Cambridge: MIT Press), 1228–1231.

[B47] Yuille A. L. Grzywacz A. M. (1989). A winner-take-all mechanism based on presynaptic inhibition feedback. Neural Comput. 1, 334–347 10.1162/neco.1989.1.3.334

